# Alcohol Consumption and Risky Drinking Patterns among College Students from Selected Countries of the Carpathian Euroregion

**DOI:** 10.1155/2018/6084541

**Published:** 2018-12-20

**Authors:** Maria Zadarko-Domaradzka, Zbigniew Barabasz, Marek Sobolewski, Edyta Nizioł-Babiarz, Beata Penar-Zadarko, Agnieszka Szybisty, Emilian Zadarko

**Affiliations:** ^1^Department of Physical Education, University of Rzeszow, Rzeszow, Poland; ^2^Department of Management, Rzeszow University of Technology, Rzeszow, Poland; ^3^Department of Medicine, University of Rzeszow, Rzeszow, Poland

## Abstract

Reduction of excessive alcohol consumption still remains a significant challenge to the actions in the scope of public health of European citizens. The aim of this study is to present the prevalence of alcohol consumption and to estimate the occurrence of risky drinking among college students from the Polish, Slovak, Romanian, and Ukrainian parts of the Carpathian Euroregion, taking social contexts into account. The consumption of alcohol was estimated on the basis of the respondents' statements regarding the quantity and frequency of their consumption of beer, wine, and vodka. The study included people from the first year of undergraduate studies. The analysis used the Chi-square independence test and odds ratios (ORs). There were significant differences in the frequency of alcohol consumption, as well as the individual types consumed, among the respondents from the analyzed countries. Of the examined college students, 70% admit to occasional drinking. The pattern of dangerous alcohol consumption occurs in the case of approximately every seventh person. Risky drinking occurs with much greater frequency among male students rather than their female counterparts. In Romania, a very small percentage of female students engage in risky drinking. The analysis did not show statistically significant differences in the frequency of risky drinking between countries. The coexistence of other adverse health behaviors, such as smoking and alcohol abuse, was confirmed.

## 1. Introduction

Alcoholic beverages, in addition to tobacco products, are the most widespread and socially accepted psychoactive substances in all age groups over 15, and their abuse is classified as harmful to health [1, 2]. Tobacco and alcohol are among the main risk factors for premature death and morbidity in Europe [3, 4]. In a 2013 ranking of the top 25 leading health risk factors in the world, tobacco and alcohol were in the 2nd and 6th place, respectively [5]. Alcohol is a substance used by adolescents in early and late puberty, causing a serious public health concern [6–8]. Health problems are not exclusively the domain of people addicted to alcohol, as they are also observed among people who abuse alcohol on a temporary or occasional basis [9]. Limits of alcohol consumption have been adopted by many countries to identify risky drinking; above these limits, people are exposed to a greater risk of experiencing detrimental health effects. The limits usually differ slightly among countries and cultures and, as in the case of Slovakia, there may be no official definition for the pure alcohol content of standard drinks [10].

The extent of alcohol consumption has social, moral, religious, and economic determinants, as described in numerous publications [11–13]. Alcohol consumption varies greatly depending on sex, age, and region [14–17]. Analysis of the literature on the subject indicates that the scale of alcohol consumption and the determinants and consequences of its abuse are areas of interest to many authors [18–23]. The results of published research confirm that dangerous alcohol consumption is observed in young adults [18, 23].

Among the research focused on risky drinking, international comparison of this behavior in students from Central and Eastern Europe has been studied the least. In 2016, a prospective study from Russia, Poland, Lithuania, and the Czech Republic was published; however, the analysis concerned people over 45 years of age [24]. National results of studies on the prevalence of alcohol consumption among students have been published, for example, in Slovakia [11], Poland [19, 25, 26], and Romania [27].

It is difficult to find reports comparing research results of alcohol drinking among students of the Carpathian Euroregion countries. The Carpathian Euroregion has existed since 1993 and covers the border regions of five countries—Poland, Romania, Slovakia, Ukraine, and Hungary. It was created to facilitate and coordinate multisector cross-border cooperation between the regions of the five postcommunist countries [28].

According to the WHO, these countries show high alcohol consumption, above the 2016 estimated European average of 10.3L per capita. Therefore, the analysis presented in this paper may supplement data on college students of the Carpathian Euroregion.

Studying cross-border health behaviors plays a key role in shaping the direction of local policy, intervention, and education of a given region in the sphere of public health and for mitigating existing threats. The aim of this study is to present the prevalence of alcohol consumption and to assess the occurrence of risky drinking among young people studying in the Carpathian Euroregion, taking into account the social context (place of residence, parents' education, and financial situation).

## 2. Materials and Methods

The analysis described in this report is based on data collected from surveys completed by a total of 1686 randomly selected college students from four significant university cities in the Carpathian Euroregion: Prešov (Slovakia), Lviv (Ukraine), Oradea (Romania), and Rzeszow (Poland). The respondents are students in their first cycle (bachelor's degree). Data from 1369 (81.2%) respondents were identified for inclusion in this cross-sectional study. The included data were taken from surveys that provided comprehensive information on alcohol consumption and other variables included in the analysis. Incomplete questionnaires were not considered in the analysis (a filled-in profile, but lack of answers to the questionnaire questions or the opposite, a missed answer to at least one question or the answer marked in a way not compliant with the instructions). The age range of the studied population is 18–22 years. The gender structure of the respondents in the compared countries is presented in Table 1.

The survey used a traditional pencil-and-paper (p&p) questionnaire in the mother tongue of the country being analyzed, and it was completed voluntarily and anonymously. Each questionnaire contained information for the participant about the purpose of the study. Questions related to alcohol consumption were of a closed type; additional variables such as age, sex, parents' education, place of residence, financial situation during studies, and cigarette smoking were also included. The tool had been verified in terms of the comprehensibility of the questions formulated, demonstrating the absence of linguistic and structural failures. The project was approved by the Bioethical Commission of the University of Rzeszow in Rzeszow (Poland) (No. 1/06/2014). The amount of alcohol consumed was estimated on the basis of information obtained from the respondents on the frequency of consumption of alcoholic beverages (vodka, beer, and wine), including one-off consumption, in the last year. The quantity–frequency (QF) method has been used in this study of drinking consumption. The frequency of drinking was measured as follows: I do not drink; I drink several times a year; once a month; once a week; 3–4 times a week; every day. The amount of alcohol consumed was measured according to portions defined as 250 mL of 5% beer, 30 mL of 40% vodka, or 100 mL of 12% wine, each of which being one standard portion of pure alcohol, about 10 g. A graphical depiction of the different portions of ethanol for the three measured alcohol varieties was presented to and discussed with the respondents before they completed the questionnaire. Consumption of excessive amounts of alcohol at once or over a period of time, without entailing negative somatic or mental consequences, is referred to as risky drinking [29, 30]. Risky drinking may also include situations of drinking alcohol under inappropriate circumstances, such as during pregnancy [31] or when taking medications that interact with alcohol [32]. According to the Polish drinking guidelines, which coincide with the limits set by the WHO, the limit for women is 20–40 g of pure alcohol (2–4 standard drinks) per day, and the limit for men is 40–60 g (4–6 standard drinks) [33]. Based on the WHO classification [33] of alcohol consumption patterns, risky drinking was defined as drinking at least 140 g of alcohol per week for women (20 g daily) and 280 g per week for men (40 g per day). The analysis took into account social variables (parents' education, permanent place of residence, residence during studies, and financial situation during studies) and those related to alcohol consumption and smoking (frequency of consumption, types of alcoholic beverages consumed, and smoking status). The comparisons were made based on the gender and country of the students surveyed.

Demographic data and the pattern of alcohol consumption are presented using descriptive statistics. In the analysis reported below, the Chi-square independence test was used for (1) analyzing the significance of differences in the distribution of traits describing the consumption of particular types of alcohol among countries and (2) demonstrating the relationship between risky drinking and independent factors. The comparison of risky drinking frequency between women and men was supplemented with the odds ratio (OR). The chance of the occurrence of a given phenomenon is determined by the result of dividing the probability of its occurrence by the probability of the occurrence of the opposite event. For example, if a given phenomenon occurred in the analyzed cases, the chance of its occurrence is 1. The odds ratio is a comparison of such a defined level of chance between two groups. To assess whether the difference in the frequency of the occurrence of a given phenomenon is statistically significant, the 95% interval for the OR is also given. If this interval does not include the value 1, it can be concluded that there is a significant difference in the chances of a given phenomenon occurring between the two compared groups. Statistical significance was assumed for p values < 0.05. Statistical analyses were performed using the STATISTICA v. 12 program.

## 3. Results 

### 3.1. Detailed Information about Drinking Alcohol in the Studied Population

The information from the study population's self-assessment shows significant differences in the frequency of alcohol consumption as well as in the frequency of individual types consumed among respondents from different countries, as shown in Table 2.

Of the surveyed college students, 70% consumed alcohol occasionally. The largest percentage (41.2%) of the respondents who stated that they did not drink alcohol were Romanian students. Most participants from Poland drank alcohol (82.3%) occasionally, while the statements from the other nations were equally high, with over 50% drinking alcohol occasionally. Every day, beer was said to be consumed by 4.4% of Slovak and 3.7% of Ukrainian participants. Almost one-fourth of the students from Poland and Ukraine drank beer 3–4 times a week, while 5.3% of Slovaks and 5.5% of Poles admitted to drinking vodka several times a week. Among the surveyed students from Ukraine, 1.4% stated that they drank vodka daily. Romanian students most often drank wine—0.9% daily and 7.8% 3–4 times a week. The students from Poland and Slovakia drank the least amount of wine. The distribution of the frequency of alcohol consumption by women and men in individual countries is presented in Table 3. The smallest differences between men and women (in particular, regarding wine consumption) occur in the Ukrainian group of students.

### 3.2. Risky Drinking

The pattern of risky alcohol consumption occurred in the case of 13.6% of people from the studied group.

Taking into account the gender of the respondents, it was found that this phenomenon occurred at a much greater frequency among male college students. That is, men consumed alcohol in a risky manner more often than female college students did (OR = 2.50, 95% CI = 1.81–3.46) (see Table 4).

The frequency of risky drinking among female and male college students was also considered independently for the population from each country (Table 4). Only in Ukraine are there no statistically significant differences in the frequency of risky drinking among female and male students (p = 0.4228). In contrast, in the other countries, a significantly higher percentage of men drank in a risky manner. More than twice as many male respondents from Poland (OR = 2.22, 95% CI = 1.30–3.80) and Slovakia (OR = 2.33, 95% CI = 1.29–4.18) and almost six times as many from Romania (OR = 5.88, 95% CI = 2.45–14.08) were more likely to engage in risky drinking, compared to the surveyed female students from these countries. In Romania, a small percentage of female students drank in a risky way. The results of the comparative analysis of the occurrence of risky drinking among students from individual countries indicate a lack of statistically significant differences between the countries in the incidence of risky drinking among male college students. In contrast, for female students, the difference between countries is statistically significant (p = 0,02), although it boils down to differences only for Romania—in this country, the level of risky drinking among female students is lower than in the other countries.

### 3.3. Factors related to Risky Alcohol Drinking

Table 5 shows the percentage of people who drank in a risky manner at different levels of several independent factors, i.e., place of permanent residence, residence during studies, parents' education, financial situation during studies, and cigarette smoking. The analysis was carried out separately for each country and sex. A key variable associated with risky alcohol consumption by students is smoking cigarettes. This relationship is present for both female (p < 0.01) and male college students (p = 0.02) in Poland and Slovakia (p < 0.001, p < 0.001) and only for male students from Ukraine (p < 0.01) and Romania (p < 0.001).

The other analyzed variables for the group from Slovakia, Ukraine, and Romania do not have a clear relationship with the risky drinking model. In contrast, in Poland, there is a relationship between the place of residence and risky drinking among female students, more so among urban residents (p = 0.04). There is also a dependence between the place of residence during studies and a pattern of risky drinking among male students, definitely more so among students living in dormitories (p = 0.01). The conducted analysis also shows a significant relationship between father's education and risky drinking among female participants. Female college students whose fathers are better educated are more likely to drink in a risky way (p = 0.02).

## 4. Discussion

Excessive alcohol consumption is a recurring phenomenon among students of many universities in the world [34–36]. In both Canada and Australia, risky drinking is associated with the ages of 18–24 years [13]. Research among the Swedish population showed that the 18–29 age group accounted for almost one-third of people who drink dangerously [18]. Other reports indicate that the rates of risky drinking and heavy episodic drinking in young people, especially women, have increased in many OECD countries [10]. Also, the results of a study published at the beginning of 2017, comparing alcohol consumption for the periods 2001–2002 and 2012–2013, drew attention to the increase in the percentage of people who drink riskily in the American population [23].

College students are a special group of young adults as far as alcohol consumption is concerned. Factors such as increased independence, reduced parental supervision, and more social contacts potentially contribute to the increased consumption of alcohol in this group [37]. Research results have highlighted the vulnerability of these young adults, university students, to risky health behaviors [22, 27, 34, 38–44].

According to the present study, over 80% of the college students in the Carpathian Euroregion consume alcohol, of which 70% admit to its occasional consumption, and 5.2% consume it regularly. Beer is the most preferred drink for all students. Gender is a factor that significantly differentiates both alcohol consumption and the type of alcohol among subjects. The distribution of the alcohol consumed by participants from Poland indicates that this group drinks more beer and vodka than those from the other three countries, but it consumes less wine than the respondents from Slovakia and Ukraine, which agrees with the reports of the WHO [45], according to which, Polish citizens consume most of all beer, followed by vodka (spirits) and wine. The research of Podstawski et al. [26] proved that beer remains the most popular alcoholic beverage among Polish students. Similarly, the data on the type of alcohol preferred by the Romanian students are in line with the WHO data [45], which state that the highest percentage of Romanian people drink beer, followed by wine and vodka. Students from Slovakia and Ukraine consume spirit drinks the least, preferring the other two types of alcohol. Beer and wine predominated, which does not fully coincide with the WHO countrywide data for these countries. At the same time, the gender classification shows that male college students from Slovakia and Ukraine consume the most beer, followed by vodka and wine. On the other hand, female students from Romania, Slovakia, and Ukraine more often choose wine than beer or spirits, which agrees with reports by other authors [14, 27]. When analyzing the results, one should consider the regional differences related to the culture of drinking alcohol [46]. Our data for college students from Poland and Ukraine are not fully in line with the trends characteristic of the drinking culture of the region (the northern European pattern); these countries are regarded as postcommunist, where the preferred type of alcoholic drinks are spirits.

Risky drinking occurs with a much greater frequency among men than women, which is in accordance with previous studies [11, 47–49]. An important reason for this is likely related to cultural and moral considerations. In contrast to women, men are more likely to engage in risky behaviors and dangerous activities. In a cross-sectional study of the university students of the Russian Federation, men were more likely to consume alcohol and drank much more than women [50]. Studies of Romanian youth also showed a higher frequency of alcohol consumption among men compared to women [14]. However, there are reports that attest to college women getting drunk more frequently than men [51]. Our study shows that, in Romania, a very small percentage of women drank in a risky way. As a researcher of alcohol consumption in Romania, Rada [14] pointed out that in Romanian society, in general, women are more criticized than men for smoking or consuming alcoholic beverages in public places. Our research also noted an exception. The analysis of a sample of students from Ukraine did not show statistically significant differences in the frequency of risky drinking among women and men (p = 0.42) from this country. According to national research conducted in Ukraine, an age of 18–25 is the main risk factor for alcohol consumption [52]. There are studies showing that women are more diverse than men in terms of alcohol consumption [53].

Our study did not show statistically significant differences in the frequency of risky drinking between the analyzed countries, with the exception of Romanian students. In the studied group of students, the percentage of men engaging in risky drinking is similar across the four compared countries of the Carpathian Euroregion (21.1% in Poland, 20.1% in Slovakia, 17.6% in Romania, and 16.1% in Ukraine). Similar to international studies [35], students from Poland and Slovakia are characterized by higher alcohol consumption (heavy drinking) relative to the other countries covered by this study. The highest percentages of women who are risky drinkers are in Ukraine (12.2%), Poland (10.8%), and Slovakia (9.8%). The lowest percentage of such women is recorded for Romania (3.5%), which is confirmed by results from other studies [35]. In our study, interestingly, there is a lack of such distinct differences for the percentage of risky drinkers between countries (especially in men) in comparison with the statistically significant differences in the frequency of consumption of particular types of alcohol. In other words, although the preferences for consuming specific types of alcohol are significantly different in the compared countries, the percentage of people who drink alcohol in a risky manner occurs to a similar extent, regardless of the type of the drink consumed.

The result of the present research indicates a relationship between smoking and drinking alcohol in a risky manner, which corresponds to the results of other authors' work [34, 49]. The cooccurrence of behaviors such as smoking and alcohol abuse carries increased risks of cardiovascular complications and cancer, which are serious threats to public health [54].

International studies on intensive drinking among college students have shown that drinking alcohol in large quantities is associated with well-educated parents and family wealth [35]. Some studies report a significant relationship between problematic alcohol consumption and a higher level of fathers' education [55]. In our study, only in relation to Polish female students there was a significant correlation between higher education of the father and a good financial situation during school studies and risky alcohol consumption. In turn, there was a connection between risky drinking and a difficult financial situation (p < 0.01) for female students from Ukraine. Ukraine is not a member of the European Union (EU) and is a country with a low national income per capita; however, alcohol is easily available there at a low price relative to earnings. Studies on the socioeconomic status (SES) of Ukrainian residents and its relation to alcohol consumption have shown that younger people with low status (SES) have higher rates of alcohol consumption and smoking [21]. In the other countries studied here, these variables do not show any relation to the risky drinking model.

Studies have shown that intensive drinking is more common among students who live on campus [50], away from home [35]. A study of Slovak students reported that life with parents during the semester was consistently associated with less frequent episodic drinking, fewer episodes of drunkenness, and fewer problems with drinking [11]. In contrast to the studies quoted above, no significant relationship was found between the place of residence during studies and a risky pattern of alcohol consumption by students from the studied countries. Male college students from Poland are the only exception (p = 0.01). Students living in a dormitory drink in a risky way more often.

## 5. Limitations

The nature of this study includes some limitations that should be considered when interpreting the results. The limitation is the lack of data from the Hungarian part of the Carpathian Euroregion. The sample included students in their first stage of studies (bachelor's degree). Therefore, generalization of the findings to all students should be taken with caution. Further studies accounting for these limitations are necessary.

## 6. Conclusions

Ethyl alcohol is a very popular psychoactive drug among young people, the abuse of which often coexists with other risky behaviors. In the studied academic environments of the Carpathian Euroregion, it was found that the risky drinking model occurs to a similar degree, regardless of the country of origin. The study of health behaviors in cross-border areas plays a key role in shaping the direction of local policy, intervention, and education of a given region with respect to the sphere of public health and mitigation of existing threats. The results of this research can be used in planning regional preventive programs in the academic environment, covering the countries of the Carpathian Euroregion.

## Figures and Tables

**Table 1:** Structure of the studied group.

Gender	Country								Total	
	Poland		Slovakia		Ukraine		Romania			
	N	%	N	%	N	%	N	%	N	%
Female	223	51.1	215	56.0	90	42.1	199	59.4	727	53.1
Male	213	48.9	169	44.0	124	57.9	136	40.6	642	46.9
Total	436	100.0	384	100.0	214	100.0	335	100.0	1369	100.0

**Table 2:** Self-assessment of the frequency of drinking different types of alcohol by country.

	Poland		Slovakia		Ukraine		Romania		Total		p value
	N	%	N	%	N	%	N	%	N	%	
Alcohol											< 0.001
never	34	7.8	52	13.5	44	20.6	138	41.2	268	19.6	
in the past	13	3.0	26	6.8	21	9.8	10	3.0	70	5.1	
occasionally	359	82.3	287	74.7	135	63.1	179	53.4	960	70.1	
regularly	30	6.9	19	4.9	14	6.5	8	2.4	71	5.2	
Standard drinks per week mean (SD)	10.2 (14.1)		9.7 (16.1)		9.7 (14.7)		6.5 (14.6)		9.1 (14.9)		

Beer											< 0.001
I do not drink	64	14.7	126	32.8	67	31.3	169	50.4	426	31.1	
several times a year	31	7.1	32	8.3	7	3.3	32	9.6	102	7.5	
once a month	81	18.6	39	10.2	19	8.9	20	6.0	159	11.6	
once a week	140	32.1	100	26.0	58	27.1	62	18.5	360	26.3	
3–4 times a week	107	24.5	70	18.2	55	25.7	44	13.1	276	20.2	
every day	13	3.0	17	4.4	8	3.7	8	2.4	46	3.4	
Standard drinks per week mean (SD)	6.6 (9.4)		5.0 (8.3)		5.8 (9.0)		3.7 (9.3)		5.3 (9.1)		

Vodka											< 0.001
I do not drink	92	21.1	148	38.5	90	42.1	226	67.5	556	40.6	
several times a year	104	23.9	61	15.9	24	11.2	38	11.3	227	16.6	
once a month	130	29.8	67	17.4	46	21.5	21	6.3	264	19.3	
once a week	85	19.5	84	21.9	44	20.6	41	12.2	254	18.6	
3–4 times a week	24	5.5	24	6.3	7	3.3	8	2.4	63	4.6	
every day	1	0.2	0	0.0	3	1.4	1	0.3	5	0.4	
Standard drinks per week mean (SD)	2.6 (5.9)		2.8 (6.5)		2.4 (6.4)		0.9 (3.7)		2.2 (5.7)		

Wine											< 0.001
I do not drink	157	36.0	129	33.6	74	34.6	162	48.4	522	38.1	
several times a year	117	26.8	71	18.5	42	19.6	50	14.9	280	20.5	
once a month	82	18.8	88	22.9	42	19.6	35	10.4	247	18.0	
once a week	69	15.8	71	18.5	46	21.5	59	17.6	245	17.9	
3–4 times a week	9	2.1	24	6.3	8	3.7	26	7.8	67	4.9	
every day	2	0.5	1	0.3	2	0.9	3	0.9	8	0.6	
Standard drinks per week mean (SD)	1.0 (2.3)		1.9 (5.3)		1.4 (3.9)		1.9 (5.3)		1.5 (4.4)		

**Table 3:** Frequency of alcohol consumption with reference to gender and country.

	Poland (%)		Slovakia (%)		Ukraine (%)		Romania (%)		**p**_F_/**p**_**M**_^1^

Frequency of consumption of	Female	Male	Female	Male	Female	Male	Female	Male	

Alcohol									< 0.001/< 0.001
never	9.4	6.1	15.8	10.7	24.4	17.7	55.8	19.9	
in the past	1.8	4.2	3.3	11.2	3.3	14.5	2.5	3.7	
occasionally	88.8	75.6	78.6	69.8	68.9	58.9	40.2	72.8	
regularly	0.0	14.1	2.3	8.3	3.3	8.9	1.5	3.7	
p_F vs. M_^2^	< 0.001		< 0.001		0.01		< 0.001		

Beer									< 0.001/0.03
I do not drink	18.4	10.8	42.3	20.7	40.0	25.0	67.8	25.0	
several times a year	11.2	2.8	11.6	4.1	3.3	3.2	11.1	7.4	
once a month	26.9	9.9	10.7	9.5	10.0	8.1	3.5	9.6	
once a week	32.7	31.5	26.0	26.0	28.9	25.8	11.1	29.4	
3–4 times a week	10.8	39.0	8.4	30.8	16.7	32.3	5.0	25.0	
every day	0.0	6.1	09	8.9	1.1	5.6	1.5	3.7	
p_F vs. M_	< 0.001		< 0.001		0.04		< 0.001		

Vodka									< 0.001/< 0.001
I do not drink	22.9	19.2	45.6	29.6	60.0	29.0	78.4	51.5	
several times a year	34.5	12.7	22.3	7.7	10.0	12.1	10.6	12.5	
once a month	31.4	28.2	18.6	16.0	16.7	25.0	4.5	8.8	
once a week	8.5	31.0	12.1	34.3	11.1	27.4	6.0	21.3	
3–4 times a week	2.7	8.5	1.4	12.4	2.2	4.0	0.5	5.1	
every day	0.0	0.5	0.0	0.0	0.0	2.4	0.0	0.7	
p_F vs. M_	< 0.001		< 0.001		< 0.001		< 0.001		

Wine									< 0.001/< 0.001
I do not drink	29.1	43.2	27.4	41.4	31.1	37.1	59.3	32.4	
several times a year	32.7	20.7	23.7	11.8	21.1	18.5	18.1	10.3	
once a month	23.3	14.1	30.2	13.6	18.9	20.2	7.0	15.4	
once a week	12.6	19.2	14.0	24.3	25.6	18.5	12.6	25.0	
3–4 times a week	2.2	1.9	4.2	8.9	3.3	4.0	3.0	14.7	
every day	0.0	0.9	0.5	0.0	0.0	1.6	0.0	2.2	
p_F vs. M_	< 0.001		< 0.001		0.63		< 0.001		

^1^ **p**_F_/**p**_**M**_: assessment of the significance of differences between the frequency of alcohol consumption in individual countries (separately for female and male groups).

^2^ **p**_F  vs.  **M**_: assessment of the significance of differences between the frequency of alcohol consumption between female and male groups (separately for each country).

**Table 4:** Risky drinking among respondents, including gender and country.

	Female (%)	Male (%)	Odds ratio (95% CI)	p value
Total	8.7	19.2	2.50 (1.81–3.46)	< 0.001
Poland	10.8	21.1	2.22 (1.30–3.80)	< 0.01
Slovakia	9.8	20.1	2.33 (1.29–4.18)	< 0.01
Ukraine	12.2	16.1	1.38 (0.63–3.05)	0.42
Romania	3.5	17.6	5.88 (2.45–14.08)	< 0.001

**Table 5:** Factors associated with risky drinking.

	Poland (%)		Slovakia (%)		Ukraine (%)		Romania (%)	
	Male	Female	Male	Female	Male	Female	Male	Female
Factors								

Place of residence								
Village	22.1	7.1	26.7	7.7	12.2	16.7	11.5	6.4
City	19.2	15.8	14.9	12.5	18.5	10.2	21.4	1.8
**p value**	0.60	0.04	0.06	0.24	0.37	0.38	0.14	0.09

Living during studies								
family house	17.4	6.1	17.2	4.8	16.0	12.5	17.4	1.1
homestay	16.1	13.6	11.1	7.7	13.8	10.7	18.2	4.8
dormitory	40.0	16.7	24.0	14.0	17.8	13.6	17.6	6.1
**p value**	0.01	0.11	0.23	0.13	0.90	0.95	1.00	0.26

Father's education								
vocational	23.8	5.6	16.4	8.2	16.7	5.0	14.6	3.1
secondary	17.6	16.7	22.4	9.1	23.8	29.4	16.4	2.2
higher	17.6	20.0	22.6	14.3	14.9	10.2	25.8	8.1
**p value**	0.54	0.02	0.64	0.54	0.64	0.06	0.41	0.25

Mother's education								
vocational	24.5	8.8	15.4	5.5	11.5	10.0	10.3	3.7
secondary	20.2	9.8	23.6	12.7	27.3	15.4	17.9	1.9
higher	17.6	17.5	17.5	7.1	16.3	10.9	25.6	7.7
**p value**	0.68	0.31	0.50	0.27	0.50	0.88	0.21	0.25

Smoking								
never	16.2	6.8	10.9	3.9	7.2	8.0	8.8	3.3
in the past	28.6	20.7	20.7	5.9	5.6	15.8	46.7	7.1
occasionally	10.0	19.4	37.5	14.3	58.3	25.0	30.0	0.0
regularly	46.2	11.1	38.7	35.5	37.5	40.0	55.6	7.7
**p value**	< 0.01	0.04	< 0.01	< 0.001	< 0.001	0.15	< 0.001	0.59

Smoking^1^								
no	16.2	6.8	10.9	3.9	7.2	8.0	8.8	3.3
yes	29.7	18.9	31.6	18.6	30.4	21.9	44.1	4.3
**p value**	0.02	< 0.01	< 0.001	< 0.001	< 0.01	0.07	< 0.001	0.73

Financial situation								
difficult	15.0	5.7	0.0	0.0	25.0	41.7	24.1	6.1
average	22.2	4.7	21.9	6.3	11.8	7.4	12.5	3.6
good	22.4	17.6	20.6	11.0	17.5	7.8	19.6	2.7
**p value**	0.59	0.01	0.45	0.50	0.42	< 0.01	0.37	0.65

^1^Dichotomous division, where “yes” contains smoking in the past and currently.

## Data Availability

The data used to support the findings of this study are available from the corresponding author upon request.
